# *ForAlexa*, an online tool for the rapid development of artificial intelligence skills for the teaching of evolutionary biology using Amazon’s Alexa

**DOI:** 10.1186/s12052-022-00169-z

**Published:** 2022-06-27

**Authors:** Luan Pinto Rabelo, Davidson Sodré, Marcelo Soares dos Santos, Caio César Silva Lima, Stephen F. Ferrari, Iracilda Sampaio, Marcelo Vallinoto

**Affiliations:** 1 Laboratório de Evolução, IECOS, Universidade Federal do Pará, Campus de Bragança, Bragança, Brazil; 2 Centro de Investigação em Biodiversidade and Recursos Genéticos, CIBIO-InBIO, Universidade do Porto, Porto, Portugal; 3 Universidade Federal Rural da Amazônia (UFRA), Campus de Capitão Poço, Capitão Poço, Brazil; 4 Universidade Federal do Maranhão, Campus Bom Jesus, Imperatriz, Brazil; 5 Laboratório de Ecologia da Conservação, Universidade Federal de Sergipe, São Cristovão, Brazil

**Keywords:** Intelligent personal assistants, Social isolation, Teaching, Evolution

## Abstract

Intelligent Personal Assistants (IPAs), such as Amazon’s Alexa, are now widely used for an ample variety of tasks, ranging from personal management to education. These tools have shown considerable promise for student-educator interactions, especially at a distance, a potential that has come to the forefront during the ongoing COVID-19 pandemic. Even so, this potential is still underexploited, even in the current scenario. Alexa’s apps are known as skills, which include all the different commands that Alexa is capable of executing. It is important to note, however, that the use of such technology is work-intensive and can be relatively complex. Given this, to facilitate the development of new skills in Alexa, we have developed an online tool that permits the creation of questions and answers, as well as honing the interaction between Alexa and the user. We have named this tool *ForAlexa*, which has two types of forms, Question-And-Answer (Q&A) and Random-Quote. Both these forms allow the user to build *intents* (an activity that is invoked by a spoken request from the user), but with slightly different functions. The Q&A form is used to compile answers that Alexa will offer in response to an *utterance* (question), while the Random-Quote extends the interaction between Alexa and the user, based on the questions asked in the first form. *ForAlexa* also has a help assistant, as well as a manual, which explains all the steps necessary for the design of an intent. This tool allows educators to develop apps quickly and easily for their classes and this type of app could be an alternative to be used for students with special needs, such as the visually-impaired.

**Supplementary Information:**

The online version contains supplementary material available at 10.1186/s12052-022-00169-z.

## Background

Social distancing, quarantine and the isolation provoked by the COVID-19 pandemic have forced us to modify our way of life in many different ways, which may be more or less permanent (Iivari et al. 2020). Education is one of the areas that has been most affected, and educators have been forced to rethink approaches, not only to adapt to the circumstances of the pandemic, but also in a more general context (Crawford et al. 2020; Viner et al. 2020).

One potential approach is the implementation of new techniques based on Artificial Intelligence (AI). Artificial Intelligence is now a global reality (Tan et al. 2016), which reaches an enormous number of users, worldwide, primarily through the Intelligent Personal Assistants (IPAs) found on smart devices. These IPAs include applications such as Alexa, Google Home, Siri, and Cortana (Festerling and Siraj 2020), but they have many other potential uses, including many different types of educational tools. These tools include tutorial assistants (Hales et al. 2019), the teaching of the visually, physically and hearing impaired (Garg and Sharma 2020; Samigulina et al. 2017), and personal assistants for language learning (Dizon 2017), in addition to the potential role of AI in the management of COVID-19 itself (Hallak et al. 2020; Shaikh et al. 2021). Despite these far-reaching possibilities, the potential applications of AI, and in particular IPAs, as tools for remote teaching, which has become a global priority during the COVID-19 pandemic (Basilaia and Kvavadze 2020; Dodds and Hess 2020; Viner et al. 2020), are still either relatively scarce or poorly publicized.

Alexa is one of the most widely-used IPAs, a voice-activated system developed to operate in a number of intelligent devices produced by Amazon Inc. (e.g., Amazon Echo Dot, Amazon Echo Show). However, Alexa can also be implemented in devices not produced by Amazon (Arya and Patel 2020), such as smartphones (Android, iOS), smart TVs, and IoT devices. The users of these devices can request Alexa to conduct a number of different tasks, including playing music, podcasts or audiobooks, cataloguing reminders or controlling other intelligent devices (Canbek and Mutlu 2016).

Alexa’s apps are known as skills, which include all the different commands that Alexa will be capable of executing. Each skill typically has a specific function, such as playing music from a given streaming platform or the latest headlines of a preferred news platform. A specific skill will process the user’s request for each function of this type. Like other IPAs, Alexa can be activated either by voice, with a single word or a short phrase (which can be personalized by the user) or manually, in the case of devices with a screen. Once Alexa is activated, a specific skill can be activated by voice or by a touch followed by a voice command. When a skill is requested, its content is processed by the algorithms in Alexa’s AI, such as voice recognition, machine learning, and the understanding of natural language.

**Fig. 1:**
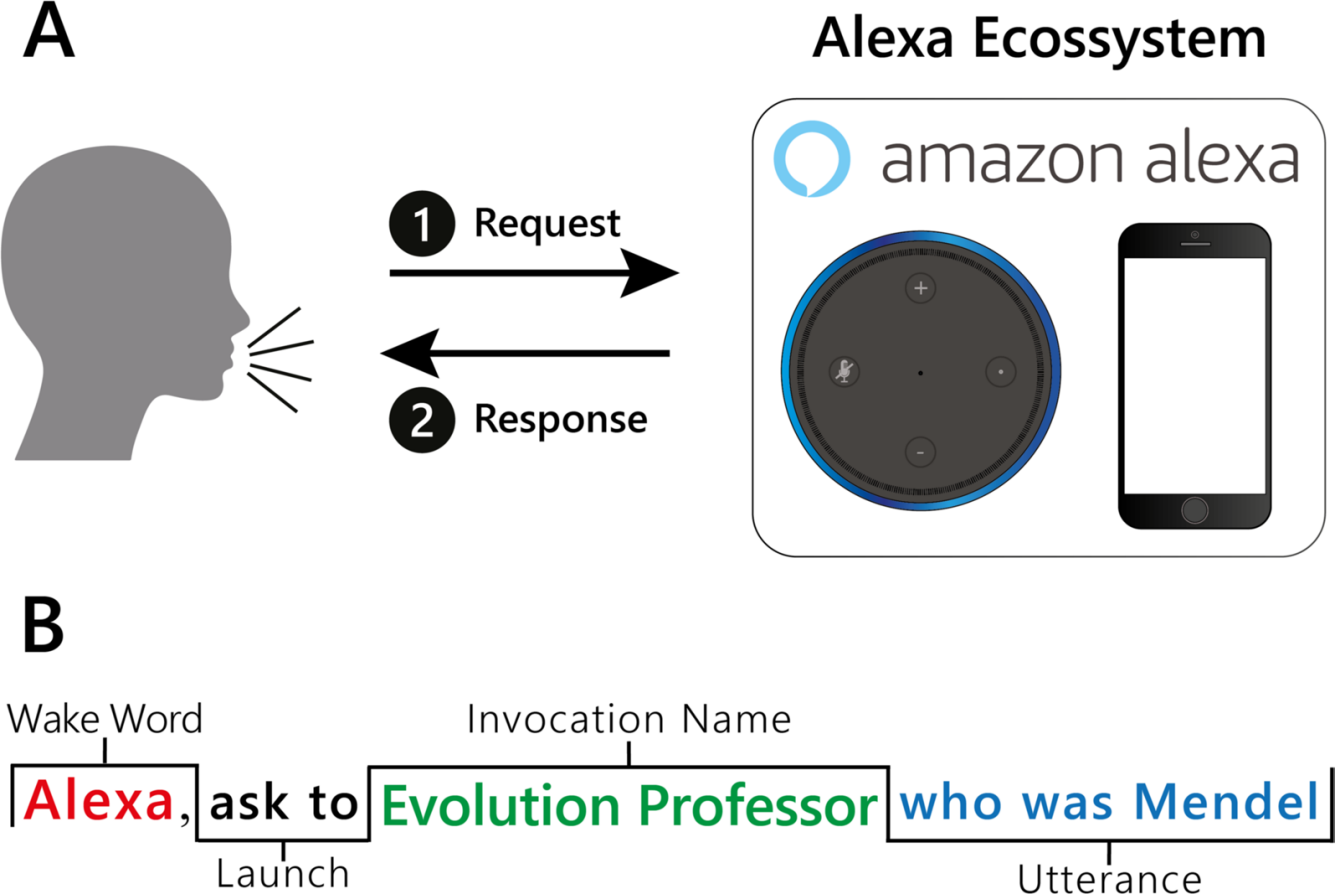
**A** Processing of the questions (user) and answers (Alexa) and **B** important concepts for the formulation of a question to be asked of Alexa

Alexa processes the user’s request by accessing web-hosted services or its own server (Amazon AWS), and then responds to the user (Fig. 1A). This response may be the execution of a task (Canbek and Mutlu 2016), which can include skills developed for a specific purpose. Devices that use Alexa have been installed in public spaces, such as museums, hospitals, and classrooms, to support tasks appropriate to different situations (Lopatovska and Oropeza 2018). It is important to note that, while Alexa, like other IPAs, is currently being tested as a teaching tool for use in the classroom (Neiffer 2018; Hales et al. 2019), there is practically no published material on the development of skills for the teaching of specific disciplines. This may be at least partly due to the difficulties of the development process, whether derived from an absence of specific knowledge on programming or a lack of computing ability on the part of the educator.

The tools would potentially be extremely valuable as auxiliary teaching aids for specific disciplines, in particular for special needs students. Even so, many of the challenges of teaching the visually impaired will still persist (Orsini-Jones 2009; Zhou et al. 2011).

To facilitate the use of this tool by other educators, in particular those that may be inexperienced in programming, we have created a web-based form page (*ForAlexa*) which explains the steps necessary for the construction of teaching skills. We also developed a skill for a specific, undergraduate-level discipline, population genetics (in Portuguese), and at the end of this discipline, we applied questionnaires to evaluate the students’ use of the skill created using *ForAlexa* .

## Methods

### Development of a skill

We chose Alexa, the artificial intelligence (AI) platform from Amazon Inc., for the present study, due primarily to the fact that the development of apps on this platform is facilitated considerably by a specific page on the Amazon system, which provides detailed instructions and examples of codes, and an extremely user-friendly interface for the development of apps, i.e., skills (Johnstone and Razavi 2018). The second reason for the choice of this IPA is that it can be used in many different types of smart device, including most types of smartphone (Android, iOS). We also considered the ongoing growth in the number of skills developed for Alexa, and the constant improvement of this AI platform (see Rausch, D. September 24th, 2020, https://developer.amazon.com/en-US/blogs/alexa/device-makers/2020/09/Build-a-More-Proactive-and-Intelligent-Smart-Home-with-Alexa).

A number of standard points are necessary for the development of a skill, such as the wake word, which activates Alexa and prepares it to receive a command from the user, and the launch words, which are used to activate the skill, and include standard terms such as “open”, “use”, and “launch”. These words antecede the invocation name, which is the name given to an interaction of a skill. To use a skill called “Evolution Professor”, for example, the user must say “Alexa, open Evolution Professor”, thus combining the launch word (Alexa) with the invocation name (Evolution Professor). The intention (*intent*) is an action that attends to a question (*utterance*) that the user asks Alexa. It is important to note here that a given intent can be activated by questions formulated in different ways. To ensure this, the developer must consider the different ways in which the user may formulate the same solicitation (see Fig. 1B).

### ForAlexa

*ForAlexa* is a web-based form and has a free source code, which is available to the community on its GitHub page (https://github.com/luanrabelo/ForAlexa).

**Fig. 2:**
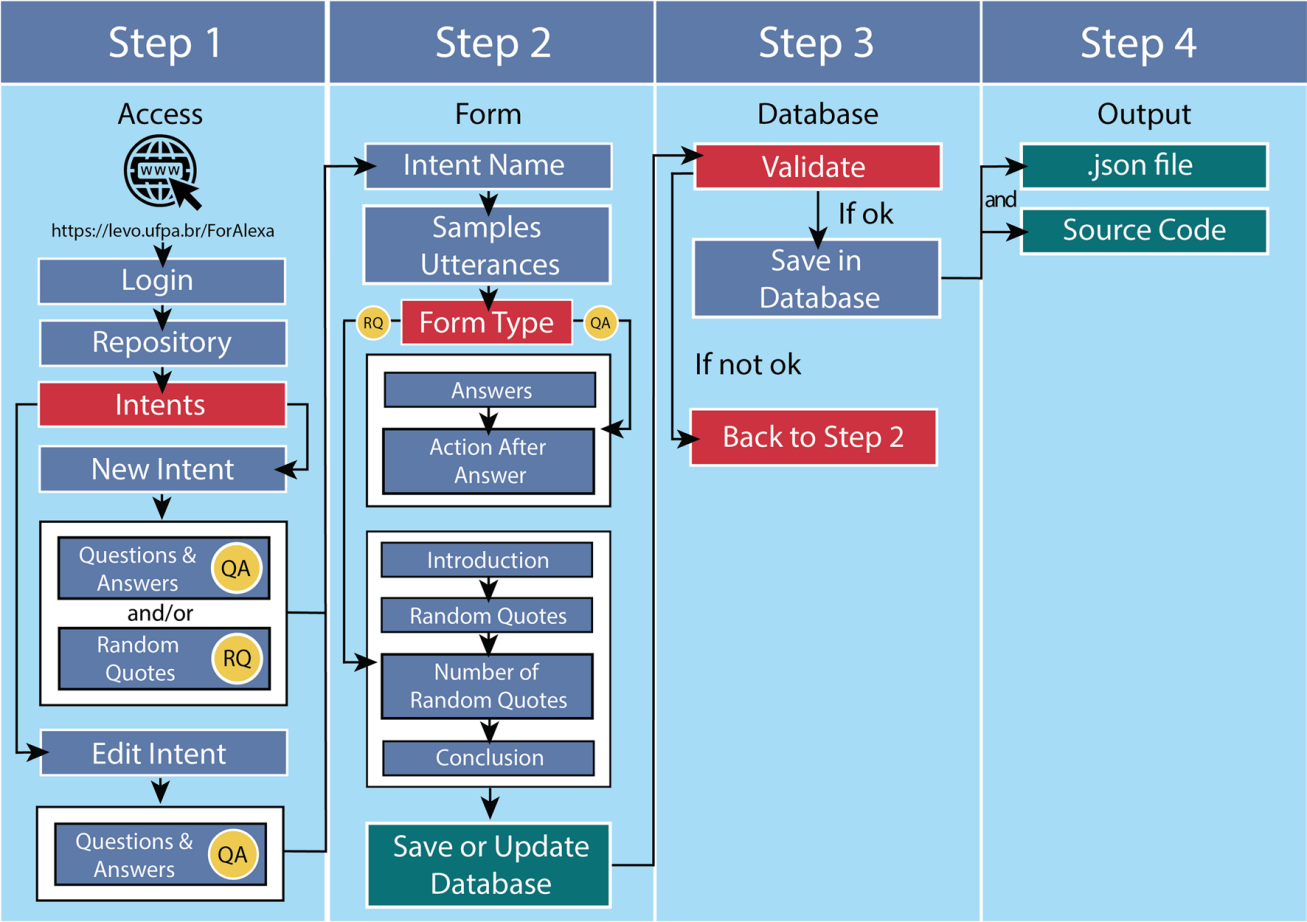
Workflow of the different options for the development of a skill based on ForAlexa. The development is divided into four steps, where the user can use only one (question-and-answer) or two types (question-and-answer and random quotes) of form, decide the level of interaction between the user and the AI, and include random questions to be asked by Alexa

To begin with, the user must register or login to the Alexa Developer Console (https://developer.amazon.com), and create a new skill (https://developer.amazon.com/alexa/console/ask), informing its name and standard language, as well as the skill model and its host. The user should then create an account and follow the instructions on the *ForAlexa* homepage. The *ForAlexa* workflow is shown in Fig. 2. *ForAlexa* has a help assistant to facilitate the steps outlined below. This assistant is turned on by default, and allows the user to understand basic concepts, what is permitted or not (letters, numbers, spaces or special characters), and even to build questions and answers rapidly, as well as reinforcing the interaction between the user and Alexa.

**Fig. 3:**
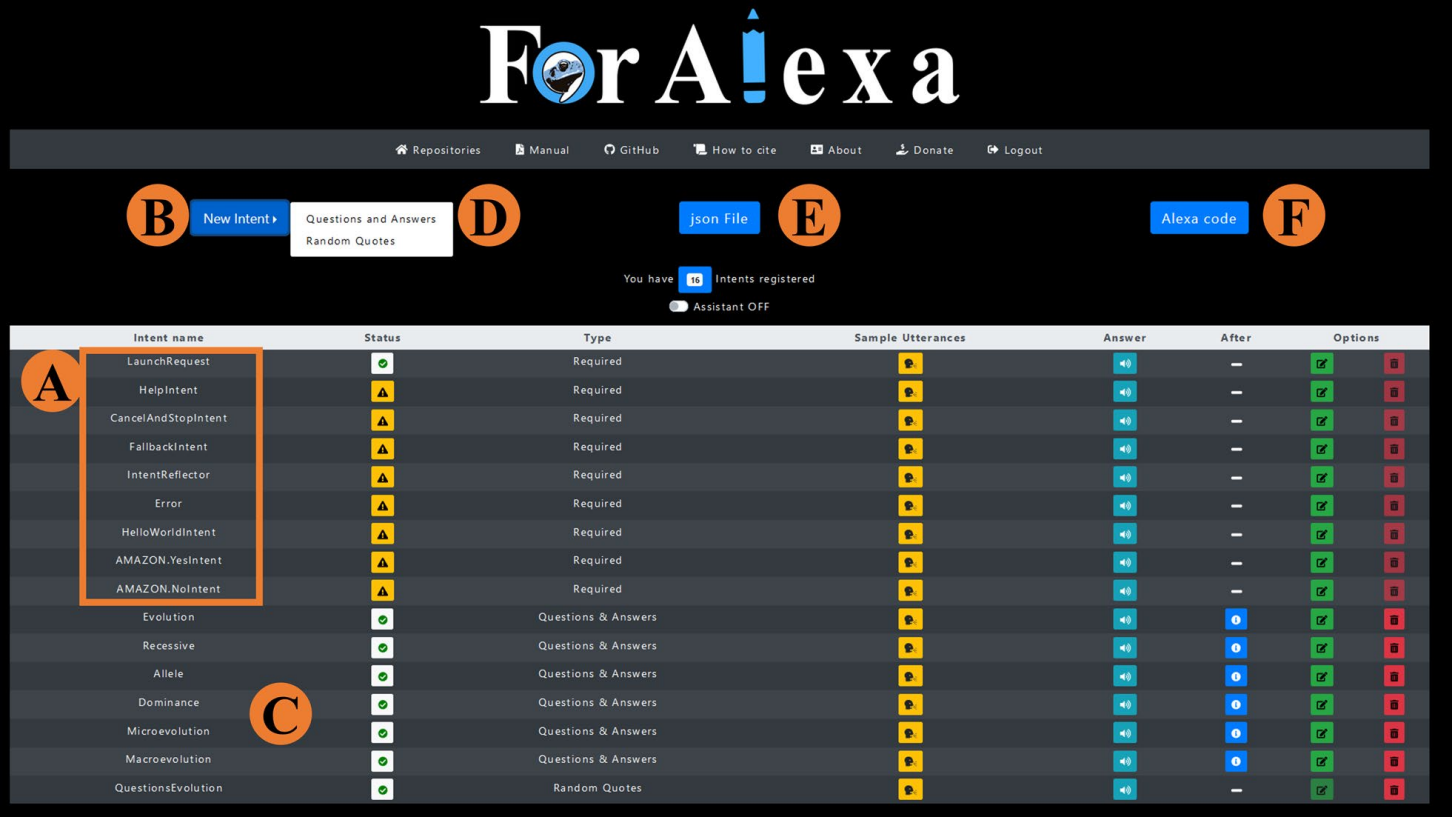
View of ForAlexa after logging in with a registered e-mail, **A** the standard required intents that can only be edited, and not deleted, and **C** a newly-created intent. To create new intents (**B**), the user must choose either question-and-answer or random quote forms (**D**, see Additional file 1: Fig. S1). Once ready, the json file (**E**) and the source code (**F**) can be generated and saved for use in a skill that exists in the Alexa platform at Amazon

Once logged in, the user must create a repository, which will present a list of required *intents* necessary for the skill to work (Fig. 3A). Once established, these *intents* cannot be deleted, only edited (although the name of a required *intent* cannot be altered). These *intents* are normally related to the invocation name of the skill (LaunchRequest) and its help request (HelpIntent). There are a number of other required *intents*, but to simplify, the *ForAlexa* help assistant only teaches how to edit the LaunchRequest (the edition of required *intents* is explained below).

Once an *intent* has been registered (Fig. 3B), it will appear at the end of the page (Fig. 3C). At this stage, the developer can edit these *intents* according to their language, with English being the default, or they can create new *intents*, choosing between Question-and-Answer (Q&A) intents or Random Quotes (Fig. 2, step 1; Fig. 3D; Additional file 1: Fig. S1). Once the type of *intent* is chosen (step 1), the corresponding form will be loaded (Fig. 2, step 2), in which the developer should inform the *intent name* and the *utterances*, that is, a name for the question (e.g., “Evolution”) and the question that Alexa must be able to answer, e.g., “What is Evolution?” (Fig. 1A). It is important to note here that as a question can potentially be formulated in a number of different ways, *ForAlexa* can include different options, e.g., “What is the Theory of Evolution?”. All the fields described above are required.

If the form is of the Q&A type, the developer must provide the answer that Alexa will provide in response to the utterance, followed by one of two options—(i) finalizing the *intent*; or (ii) asking the user a new question (*reprompt*), for example, asking whether the user requires any more information on the topic (Additional file 1: Fig. S1). In the case of a Q&A *intent* called “Microevolution”, for example, a standard *utterance* might be “What is Microevolution?”, which would be followed by an answer from Alexa and then a default *reprompt*, such as “If you want, you can ask me another question, for example, ask me questions about evolution” or (if the discipline is organized in chapters)—“If you want, you can ask me another question, for example, ask me questions on chapter one or any other chapter you like”.

If the developer wishes to include a greater level of interaction between Alexa and the user, the random-quote type of *intent* can also be included here. In this case, the form has the option of creating suggestions for random questions on a given subject or class, such as “would you like to ask more questions about evolution?” or “would you like to ask more questions about class one?”. The developer can provide to the random questions the *utterances* created in the Q&A form.

The answer field of a random quotes *intent* is substituted by the random quotes field, where the developer defines how many random quotes the AI will use, with a default value of 2. An introductory phrase (which is not obligatory), which defines the possibilities of interaction with the AI, can be introduced here. The developer then provides the random quotes that will be selected when an *intent* is activated. In the final step, the developer can provide a phrase to be spoken by Alexa following the random quote. While this phrase is not required, it does amplify the potential for interaction between the AI platform and the user. At this point, the developer can save or update the *intent* by clicking on the respective button (Additional file 1: Fig. S1).

An example of a random quotes *intent* would be: (i) *intent name* “QuestionsAboutEvolution” (no spaces allowed), and (ii) an *utterance* “Questions about Evolution”, followed by an *introduction*, such as “You can ask me”, and then all the possible questions on a given subject or class, such as “What is Evolution?”, “What is Microevolution?”, “What is Macroevolution?” and so on. Finally, in *After Alexa*, the developer may choose a sentence of the Q&A type “If you want, you can ask me another question, for example, ask me questions about Evolution”. When Alexa is asked “Questions about Evolution”, it will select questions (2 by default) randomly from the skill to ask the user, and then ask whether the user wants to ask one of these questions or whether the user wants to hear more questions chosen randomly. In other words, the dialog on a theme or a class will continue.

The first required *intent* is the LaunchRequest. Here, the developer should edit the *Skill Invocation Name*, but not the *Intent Name.* At *Skill Invocation Name*, the developer must substitute the default sentence with the name of the skill that the users of Alexa will need to say to activate the skill. This is the new name of the skill (“Evolution Professor”—see Fig. 1B). The developer must then edit Alexa’s answer to this question, for example, “Hello, welcome to the Evolution Professor skill. My name is Alexa and I will guide you through the questions in this skill. If you would like to know what questions I can answer, ask me: Questions about Evolution?” Note that there is always a question at the end of the sentence, which is the same utterance that was created in the Random Quotes form. In this case, Alexa will direct the user to ask specific questions, as described above.

The second required *intent* is the HelpIntent. Here, the default sentence—“I have no content on the topic”—can be edited, as desired, by the developer, although we would recommend leaving the default. But here, the developer must change the *Finish* option of *After Alexa* to the *Another Question* (*Reprompt*) option. In this case, Alexa will continue the interaction, saying, for example, “If you want, you can ask me another question, for example, ask me questions about Evolution?” (The default option, as presented above).

Please note that, when *intents* are registered or updated, *ForAlexa* revalidates the database (Fig. 2, step 3). This includes verifying: (i) whether an *intent name* already exists; or (ii) whether an *utterance* is duplicated in the database. Once the absence of duplications is confirmed, the data are saved, but if a duplication is found, the user is redirected to the preceding screen, which will describe the error that requires correction in step 2 (Fig. 2). The same validation process occurs in the Amazon Developer Console.

Once all the content is confirmed, the developer is directed to the next phase (Fig. 2, step 4), where all the *intents* that have been registered in the skill can be visualized, and edited or excluded. This step includes the option of generating a *json* file (Fig. 2, step 4; Fig. 3E) (which contains all the data in the format necessary for the Amazon Developer Console) and the source code (Fig. 3F). These should be saved and transferred to the Amazon page with the skill open, where the documents can be uploaded and the skill validated (consult the *ForAlexa* assistant and manual for further details).

## Results and discussion

The covid-19 pandemic has had a profound effect on all our lives, in many different ways, and Education is one of the areas that have undergone the most profound changes, with almost all activities going online (Dodds and Hess 2020; Rapanta et al. 2020). This is a novel scenario for most educators, which not only demands re-adaptation, but also the adoption of new tools, such as IPAs. Tools of this type, based on AI or the internet, have been adopted worldwide in response to the new reality provoked by the pandemic. Pacheco et al. (2020) provide an excellent example of this shift in approach, discussing the use of video calls as an alternative for the evaluation of the performance of students following anatomy classes given on a video platform.

It is important, however, not to confuse the type of skill described in the present study with the more generalist skills that are available on platforms, including Alexa. In fact, hundreds of skills can be found in the Alexa Skills Education & Reference department of both American and Brazilian Amazon Inc., for example, although they all refer generically to areas such as Biology, History, and Geography, rather than being adapted to a specific discipline or class.

The type of skill that we propose here could be used by anyone interested in the specific theme of evolution, although it is structured by the topics presented by the educator during the classes. The idea is to allow the students that participated in the classes to revise the topics presented and complement their learning (Hales et al. 2019), as well as providing certain students, such as the visually-impaired, with assistive technology (Zhou et al. 2011).

The objective of an Alexa skill developed by *ForAlexa* is to establish complementary extra-class contact between the educator and the student, which is extremely necessary in the context of the COVID-19 pandemic, that is, a forced scenario of online education (Basilaia and Kvavadze 2020; Rapanta et al. 2020).

**Fig. 4:**
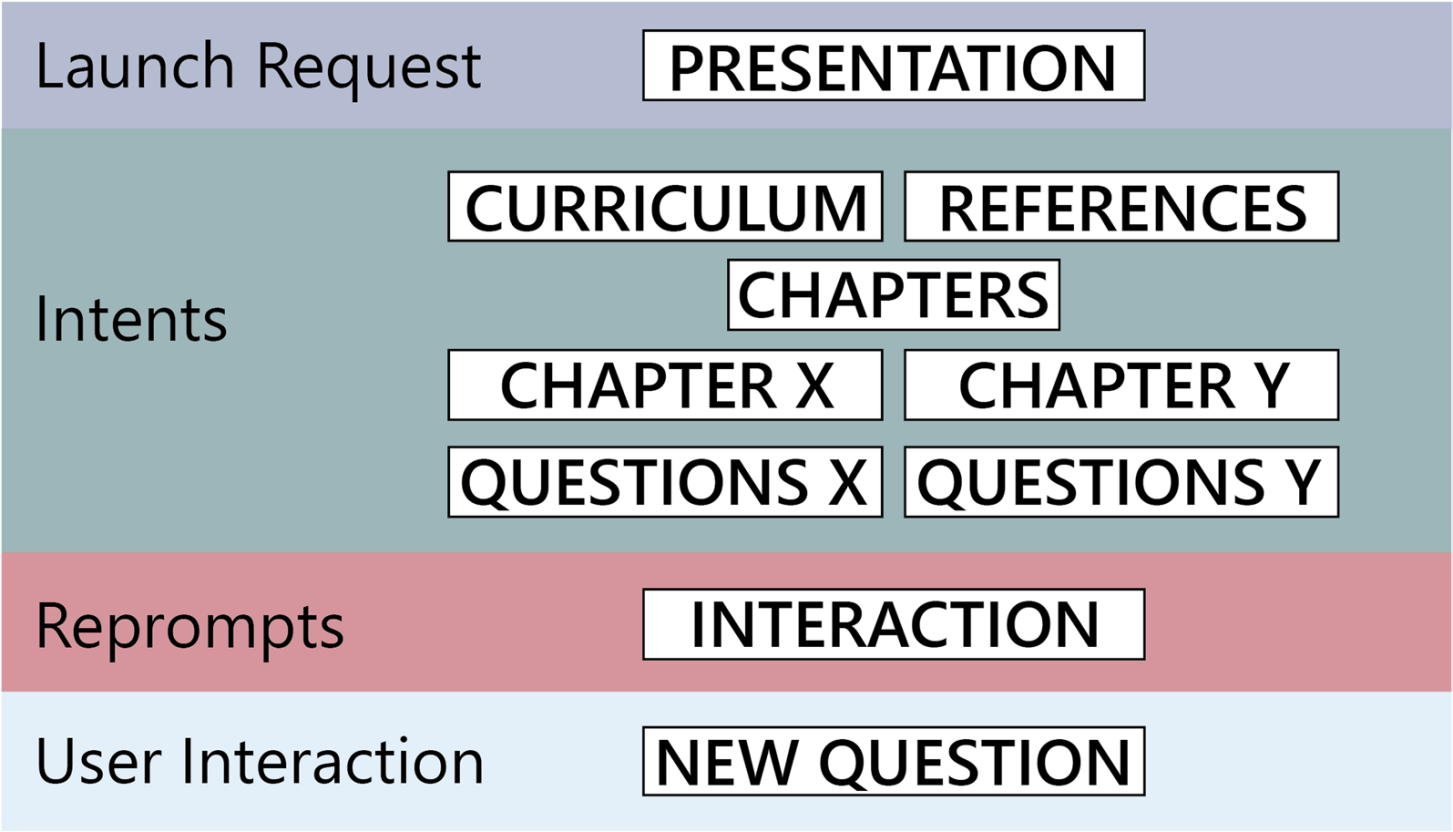
Pipeline of the organization of the skill; the presentation of the skill, the contents of the discipline and the classes, and the interaction between the question-and-answer modes of the different classes

One fundamentally important point in the creation of a skill is the Alexa–user interaction, which must ensure the most effective possible communication between the student and the skill. We bore this in mind when developing *ForAlexa*, in particular, given that the user will often ask a question inadequately (that is, in a way that Alexa cannot understand), for example, “Tell me about the Hardy-Weinberg equilibrium”, rather than “What is the Hardy-Weinberg equilibrium?”. In these cases, it is possible to include options in *ForAlexa* that allow Alexa to answer that it has no content on the topic. Alexa will then ask the user if they are interested in hearing some questions on a specific theme (HelpIntent). If the user says “yes”, the Yes.Amazon *intent* will direct the user to an alternative question. The random question form can also enable Alexa to present random questions on the subject matter (or class) and then ask the user if they would like to hear more questions. These questions are chosen randomly, but Alexa will eventually include the correct question that the developer included, i.e., What is the Hardy-Weinberg equilibrium? The organization of a skill for educational purposes is presented in Fig. 4.

This interaction with the user is fundamental, because it permits the user to establish a “conversation” with the IPA and not simply ask questions and receive answers. The idea here is for the IPA to lead the user through the skill (Moore et al. 2017), and to stop when the user either has no more questions or does not want to continue using the skill, at least for the time being. This type of interaction is fundamentally important for students with visual deficiencies (Samigulina et al. 2017), where the interaction will be the student’s guiding force when using the skill. It is important to note that these phases are especially important because this type of technology is still being refined, and requires a more detailed evaluation (Ong and Suplizio 2016), which is evolving gradually (Dizon 2017; de Barcelos Silva et al. 2020; Lopatovska and Oropeza 2018).

In addition to questions asked in the wrong format, some questions may simply not be available in the skill. In this case, it is possible to program a skill in *ForAlexa* to answer that it does not have any information on the specific point, and then to ask the user if they have any other questions. The interaction will continue in this way until the user does not want to ask any more questions.

It is also important to remember that Alexa may not always answer a question immediately, which will oblige the student to ask the same question more than once, in different ways. This is a potential problem, which demands that the developer tests the questions exhaustively in an attempt to detect the words that Alexa may misunderstand most often. This occurred most frequently in terms of the verbal or acoustic quality of the question asked by the user, i.e., the utterance (Lopatovska and Oropeza 2018), which clearly did not detract from the quality of the product (skill) to be presented to the students. Obviously, an individual may ask a question in many different ways (Kumar et al. 2018), and ultimately, if the question is not asked in a clear and adequate way, Alexa may not be able to answer.

A skill was designed in *ForAlexa* which contains questions in Portuguese on seven chapters of an undergraduate course in Population Genetics (available from the Brazilian Amazon; https://www.amazon.com.br/dp/B092G8B2YW/). While the skill does contain some more general material, which could be used by anyone interested in the theme, it is structured by the topics presented by the educator (Population Genetics) during the classes.

Once the skill has been presented to the students and its use has been demonstrated, they were free to use the skill as they prefer. It is important to emphasize, however, that the skill does not substitute the adequate study of a topic, but rather, provides a reference base for the principal concepts presented in each class. Given this, we monitored the use of the skill by the students in relation to a given topic, in terms of the questions asked and the specific use of the skill. We also asked the students to explain in their own words what they had learnt from Alexa on a specific topic.

**Fig. 5:**
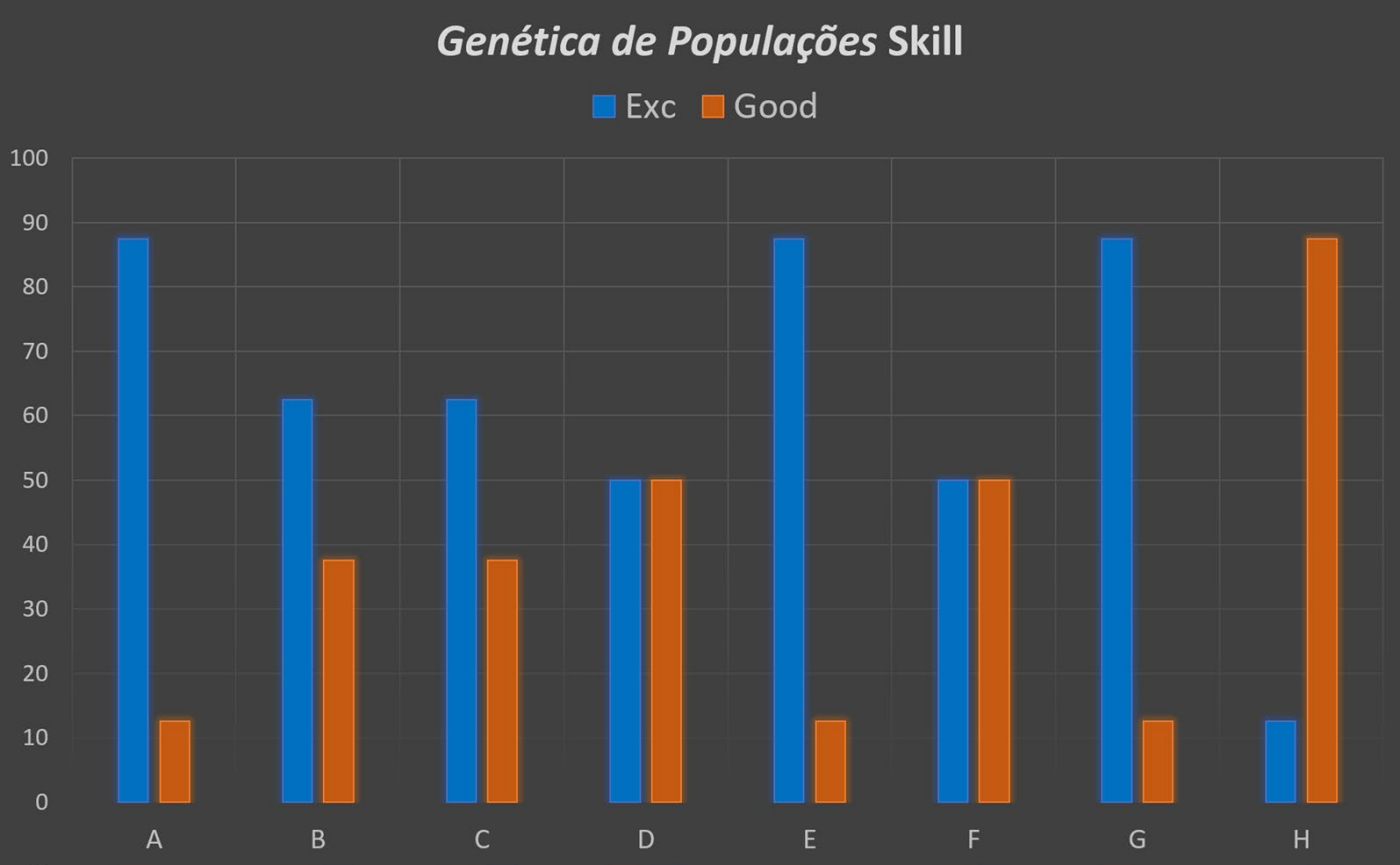
Results of the feedback from the students with regard to the questions on: **A** the purpose of the skill, **B** the consistency between the class content and the questions in the skill, **C** whether they would recommend the skill to other students; **D** their evaluation of the skill, **E** the potential of skills for other disciplines, **F** the degree to which the skill contributed to the understanding of the discipline, **G** the use of skills, like that presented here, in the teaching of the visually impaired, and **H** the capacity of the AI to understand the questions asked by the students

Overall, the Population Genetics skill was well-accepted by the students, who mostly gave it the most favorable evaluation (Fig. 5). The students responded to all the questions by evaluating Alexa’s performance invariably as either “good” (37.5% of the responses) or “excellent” (62.5%), with no evaluations in the “regular” or “bad” categories (which are thus not shown in Fig. 5). The most positive response, with the largest proportion of “excellent” evaluations, was elicited by the idea of developing the skill for the discipline, developing similar skills for other disciplines, and the use of these skill to teach visually-impaired students. The vast majority of the students (87.5%) classified Alexa’s capacity to answer the questions they asked as “good”.

The creation of a skill is an idiosyncratic task, and each educator will likely adapt it in some way to the content of their discipline and their own personal style of teaching. In our skill, for example, when Alexa is asked what the Hardy-Weinberg Equilibrium is, after providing the answer, Alexa will ask whether the user would like to know something else (as described in detail in the "Methods" section). At this point, however, the educator may wish to include more information, such as the specific section of a textbook that covers the topic, complementary websites or even direct the student to related questions. For example, Alexa could end up with a question—would you like to know what processes cause deviations from Hardy-Weinberg Equilibrium?

In the case of our skill, the only negative aspect of the use of the skill perceived by some students was the fact that Alexa did not always answer a question immediately, obliging the student to ask the same question more than once, in different ways.

It is clear that any IPA will have room for improvement, and considerable advances have been made in terms of the understanding of the structure of a language (Hirschberg and Manning 2015), although the capacity of these assistants to understand a user’s speech will almost invariably be limited in some way. This will entail certain specific problems. In the specific case of the present study, some of the students were Spanish speakers, and their diction (accent) was a predictable problem (Moore et al. 2017; Lopatovska and Oropeza 2018), which often impeded Alexa from understanding their questions. Even when the users are native speakers, there may be considerable linguistic variation, in terms of accents, ambiguous phonemes or even gender differences (Kumar et al. 2018), which may hamper Alexa’s capacity to understand a question. This problem is one of the principal challenges faced by the developers of new skills, although one potential approach is to encourage the users to better understand the mechanisms of their interaction with Alexa, and in particular, the need for clear and unambiguous pronunciation (Davie and Hilber 2018).

In the present case, the questions used for this type of skill should follow the format indicated by Alexa, and should be asked in an adequate way. As mentioned above, minor variations in the wording of the questions can be incorporated into the skill, such as “Tell me about the Hardy-Weinberg equilibrium” or “What is the Hardy-Weinberg equilibrium?”, to which Alexa will provide the same answer. It would be virtually impossible, however, to include all the potential variations on a given theme, as in the case of the more trivial questions that Alexa typically responds to, such as “what’s the weather like?” With this type of skill, then, it is necessary to construct phrases and specific wording in order to ensure an adequate response, as in the older voice-activated AI systems (Hoy 2018). Even so, as IPA technology is advancing at a fast pace, it seems likely that this problem may be resolved in the near future (Tulshan and Dhage 2018), which should guarantee the more widespread use of this type of skill.

### Utility of *ForAlexa*

The interaction between the developer and Alexa will demand a certain level of programming capacity (see Moore et al. 2017) as well as extensive and systematic testing to evaluate the effectiveness of the skill. Even though Amazon provides a user-friendly environment for the development of skills, the implementation of a personalized skill by an educator, based on specific classes, is work-intensive and can be relatively complex.

Given this, *ForAlexa* provides an excellent alternative for educators to develop their own skills for their classes. There are other useful tools in this context, such as Blueprints (Amazon), VoiceApps (www.voiceapps.com) or Voiceflow (www.voiceflow.com). While no source code is available for Blueprints and only question-and-answers in English, the latter two tools are potentially interesting, but they have only limited function in free plans.

The source code of our skill and the forms are open-access, and can be found at GitHub (https://github.com/luanrabelo/ForAlexa). After obtaining the code, the user can import the skill to their developer profile, where its different components, including its questions, answers, and invocation name, can be modified. The user can also use the online form for the rapid development of a skill. Even though the development of a skill can be facilitated, it is important to remember that many different types of problems may arise, such as the need to include special characters that are not permitted. This demands a minimum level of knowledge on the part of the developer in order to resolve problems of this type following the upload of the files to Alexa. In particular, we would emphasize, once again, the need for adequate and comprehensive testing to guarantee the creation of the most functional possible skill, which is not a simple task. For this, we would recommend any developer new to this activity to create some test questions and answers to test the efficacy of both the form and the development area in Alexa. To facilitate this, we have created a document with simple questions and answers, and all the interactions necessary for the adequate completion of the forms (see the *ForAlexa* manual).

The source code and online form represent a fundamental contribution to the rapid and efficient development of new skills for education. In support of this, a discussion forum will also be created on the GitHub page, which will include new ideas and examples for the future implementation of new skills.

In addition to these teaching possibilities, the integration of AI applications would appear to be an excellent opportunity for open-access journals to create their own skills, which would make their publications available to all potential readers, including the visually impaired.

## Conclusions

The use of skills for teaching provides the educator with excellent alternative options for their interaction with the student. Obviously, tools that can guarantee the rapid creation of skills, such as *ForAlexa*, can facilitate this task enormously.

*ForAlexa* can be used by educators of all teaching levels, and with even minimal computational capacity, to compile skills for their own teaching needs, which may even include new features that can be added by the educator for their specific needs and teaching requirements.

## Supplementary Information

**Additional file 1: Figure S1.** Forms of the (A) question-and-answer and(B) and random quotes types. An intent name (C) and utterance (D) are requiredin both forms, while an answer (E) is only required in the question-and-answertype form. The user can then finalize or manifest their interest in asking Alexaa new question (F) or ask a question (G). In the random quotes mode, the usercan choose how many random questions they would like Alexa to proffer (H), anintroductory phrase, which defines the possibilities of interaction with the AI(I), the random questions (J), add space for more random questions (K), anddefine the final phrase or question that Alexa will say (L).

## Data Availability

*ForAlexa* can be found at the following link: https://levo.ufpa.br/ForAlexa/.

## Abbreviations

AI: Artificial intelligence
IPAs: Intelligent personal assistants
